# Close or positive resection margins are not associated with an increased risk of chest wall recurrence in women with DCIS treated by mastectomy: a population-based analysis

**DOI:** 10.1186/s40064-015-1032-5

**Published:** 2015-07-10

**Authors:** Jonathan Klein, Iwa Kong, Lawrence Paszat, Sharon Nofech-Mozes, Wedad Hanna, Deva Thiruchelvam, Steven A. Narod, Refik Saskin, Susan J. Done, Naomi Miller, Bruce Youngson, Alan Tuck, Sandip Sengupta, Leela Elavathil, Prashant A. Jani, Elzbieta Slodkowska, Michel Bonin, Eileen Rakovitch

**Affiliations:** Department of Radiation Oncology, University of Toronto, Toronto, Canada; Sunnybrook Health Sciences Centre, Toronto, Canada; Institute for Clinical Evaluative Sciences, Toronto, Canada; Department of Laboratory Medicine and Pathobiology, University of Toronto, Toronto, Canada; Women’s College Research Institute, Toronto, Canada; Campbell Family Institute for Breast Cancer Research, Toronto, Canada; Department of Pathology and Laboratory Medicine, London Health Sciences Centre, London, Canada; Department of Pathology and Molecular Medicine, Kingston General Hospital, Kingston, Canada; Department of Anatomical Pathology, Henderson General Hospital, Hamilton, Canada; Department of Anatomical Pathology, Thunder Bay Regional Health Sciences Centre, Thunder Bay, Canada; Department of Pathology and Laboratory Medicine, Sudbury Regional Hospital, Sudbury, Canada

**Keywords:** Breast cancer, DCIS, Mastectomy, Radiotherapy, Surgery, Carcinoma in situ

## Abstract

Mastectomy is effective treatment for ductal carcinoma in situ (DCIS) but some women will develop chest wall recurrence. Most chest wall recurrences that develop after mastectomy are invasive cancer and are associated with poorer prognosis. Past studies have been unable to identify factors predictive of chest wall recurrence. Therefore, it remains unclear if a subset exists of women with DCIS treated by mastectomy experience a high rate of recurrence in whom more aggressive treatment may be of benefit. We report outcomes of all women in Ontario (N = 1,546) diagnosed with pure DCIS from 1994 to 2003 treated with mastectomy without radiotherapy and evaluate factors associated with the development of chest wall recurrence. Treatments and outcomes were validated by chart review. Proportional differences were compared using Chi square analyses. Survival analyses were used to study the development of chest wall recurrence in relation to patient and tumor characteristics. Median follow-up was 10.1 years. Median age was 57.1 years. 36 patients (2.3%) developed chest wall recurrence. The 10-year actuarial chest wall recurrence-free survival rates and invasive chest wall recurrence-free survival rates were 97.6 and 98.6%, respectively. There was no difference in cumulative 10 year rates of chest wall recurrence by age at diagnosis (<40 years = 5.2%, 40–44 years = 1.3%, 45–50 years = 2.9%, >50 years = 2.1%; p = 0.19), nuclear grade (high = 3.0%, intermediate = 1.4%, low = 1.0%, unreported = 2.5%; p = 0.41), or among women with close or positive resection margins (positive = 3.0%, 2 mm or less = 1.4%, >2 mm = 1.5%, unreported = 2.8%; p = 0.51). On univariate and multivariable analysis, none of the factors were significantly associated with the development of chest wall recurrence. In this population cohort, individuals treated by mastectomy experienced low rates of chest wall recurrence. We did not identify a subset of patients with a high rate of chest wall recurrence, including those with positive margins.

## Background

Ductal carcinoma in situ (DCIS), a non-invasive breast cancer, comprises about 25% of mammographically-detected breast cancers (Ernster et al. 2002). About one-third of patients diagnosed with DCIS are treated by mastectomy. Women will receive mastectomy as treatment for DCIS in cases where the disease is extensively involving the breast such that complete excision using breast-conserving surgery is not feasible or due to patient preference (Katz et al. 2005; Rakovitch et al. 2007; Chin-Lenn et al. 2013). Past studies report that individuals treated by mastectomy for DCIS experience low rates of chest wall recurrence (1–5% at 10 years) but some women will develop recurrent disease involving the chest wall (Carlson et al. 2007; Owen et al. 2013; Chadha et al. 2012; Fitzsullivan et al. 2013; Meijnen et al. 2008).

Women who develop a chest wall recurrence after mastectomy for DCIS have a poorer prognosis compared to those who do not develop chest wall recurrence (Owen et al. 2013; Chadha et al. 2012; Lee et al. 2006; Godat et al. 2009; Rashtian et al. 2008). As a result, several investigators have attempted to identify factors predictive of chest wall recurrence after mastectomy for DCIS, in an effort to identify individuals who may benefit from post-mastectomy chest wall radiotherapy, but results are inconsistent. Specifically, some studies report that women with close or involved resection margins after mastectomy have an increased risk of chest wall recurrence while others have not identified an increased risk of recurrence (Carlson et al. 2007; Owen et al. 2013; Godat et al. 2009; Chan et al. 2011; Childs et al. 2013).

Past studies are limited in their ability to evaluate factors associated with an increased risk of chest wall recurrence due to the inclusion of few patients often from a single institution (which may not be representative of the outcomes of a population of women with DCIS), inclusion of cases treated by either breast-conserving surgery or mastectomy or short follow-up intervals with subsequent few events and limited statistical power (Carlson et al. 2007; Owen et al. 2013; Chadha et al. 2012; Fitzsullivan et al. 2013; Meijnen et al. 2008; Godat et al. 2009; Rashtian et al. 2008; Chan et al. 2011; Childs et al. 2013; Kelley et al. 2011; Silverstein et al. 1995; Spiegel and Butler 2003; Vargas et al. 2005). As a result, it remains unclear if a subset of individuals with DCIS treated by mastectomy, including those with close or positive resection margins, has an increased risk of chest wall recurrence such that post-mastectomy radiotherapy might be of benefit. We report the outcomes of a large population cohort of women diagnosed with pure DCIS from 1994 to 2003 treated by mastectomy with median follow-up of 10 years. Our objective is to evaluate factors associated with the risk of chest wall recurrence after treatment by mastectomy, including the impact of close resection margins.

## Methods

### Cohort identification

The method of identification of individuals in the DCIS population cohort was previously described (Rakovitch et al. 2013). In summary, we obtained full text electronic copies of all breast pathology reports held at the Ontario Cancer Registry (OCR) from January, 1994 through December, 2003. These reports included diagnoses of DCIS, represented by International Classification of Diseases (ICD) code 233, invasive breast cancer (ICD 174), or benign conditions. Patient identifiers were removed and each case received a study ID. An automated data extraction algorithm abstracted pathology data from these reports (Currie et al. 2006). We received approximately 1,50,000 breast pathology reports. All patient identifiers were removed from the reports to ensure patient confidentiality. Cases with a final diagnosis of invasive cancer, benign disease, or DCIS with microinvasion were excluded, as were patients diagnosed with invasive breast cancer within 6 months of diagnosis of DCIS. We linked these cases with the OCR database to exclude patients with previous history of invasive cancer.

To determine each patient’s surgical treatment, we linked the study database to the Canadian Institute for Health Information (CIHI) database of hospital discharge summaries and the Ontario Health Insurance Plan database of physician billings. Surgical treatment was validated by reviewing the operative report in the primary chart. Cases treated by breast conserving surgery (with or without radiation) were excluded. The date of diagnosis is the date of the initial breast cancer surgery associated with pathological diagnosis of DCIS. We determined the number of breast surgical procedures (benign or malignant) performed by each surgeon annually through the study period using CIHI and ranked surgical volume into quintiles (lowest to highest).

### Pathology

For the current study of cases treated by mastectomy, all pathology reports were manually reviewed to confirm the diagnosis of pure DCIS and to obtain data on resection margin status and width. The following data were abstracted from original pathology reports: nuclear grade (low, intermediate, high, unreported), comedo necrosis (present, absent), multifocality (present, absent). Resection margin width was coded as ≤2 mm if DCIS was at or within 2 mm from the margin, >2 mm if the closest distance from tumor to the inked resection margin was more than 2 mm or unreported if the closest margin was not reported. Comedo necrosis and multifocality were coded as absent if not indicated in the pathology report. Tumor size was not reported for >20% of cases and therefore was not included in the analysis.

### Outcomes

We identified cases that developed chest wall recurrence after mastectomy by searching the CIHI database for surgical procedures (e.g. excisional biopsy) performed on the ipsilateral chest wall at least 6 months following mastectomy. Individuals who developed recurrent disease within 6 months of their initial DCIS diagnosis were excluded (N = 30). We identified the histological diagnosis (benign, DCIS or invasive cancer) of the recurrence by reviewing the corresponding pathology report or through linkage with the Ontario Cancer Registry and CIHI in cases where the pathology report was not available. All outcomes were determined from the date of DCIS diagnosis. Ipsilateral chest wall (invasive or DCIS) and contralateral breast recurrence are defined by the detection of invasive cancer or DCIS that developed in the ipsilateral chest wall or opposite breast, respectively, 6 months or more beyond the initial diagnosis of DCIS. The last date of follow-up is March 31, 2010. Individuals were censored at the time of their initial recurrence. Overall mortality is estimated from the date of diagnosis of DCIS to the date of death from any cause. The date of death was determined from the Registered Persons Database and cause of death from the OCR. To adjust for co-morbid illnesses we identified all diagnoses during the 12 months prior to that of DCIS as recorded in CIHI using Deyo’s method (Dunne et al. 2009).

### Statistical analyses

We studied the development of a chest wall recurrence (DCIS or invasive) as a first event in relation to patient characteristics, tumor characteristics and treatment. Cumulative incidence estimates were used to illustrate the probability of breast cancer death over time while accounting for the competing risk of other (non-breast cancer) cause of death (Dunne et al. 2009). Univariate and multivariable analyses were performed to examine the effect of each covariate using Cox proportional hazards model. The hazard ratios resulting from this semi-parametric approach were used as measures of association between each risk factor and the outcomes. In subgroup analyses, non-parametric Kaplan–Meier method was used to estimate the survival times of groups and log rank test was used to test for equality between groups. In accordance with institutional policy, to avoid the risk of identity disclosure and risk patient confidentiality we were unable to report cell size for cell counts between 1 and 5. Cell counts between 1 and 5 are reported as ≤5.

## Results

We identified 5,322 women diagnosed with pure DCIS treated from 1994 to 2003; of these, 1,821 were treated with mastectomy. Patients were excluded if they received post-mastectomy radiation (N = 163), had evidence of lymph node metastases (N = 82) at the time of diagnosis, developed invasive breast cancer within 6 months of diagnosis (N = 30) or change in diagnosis after pathology review (N = 22) because our objective was to evaluate the outcomes of individuals with pure DCIS treated by mastectomy alone. The study population includes 1,524 individuals. The mean age was 57.1 years. 426 patients (28.0%) had high grade DCIS, 668 (43.8%) had margins ≤2 mm, 436 individuals (28.6%) had negative resection margins >2 mm, and 340 (22.3%) patients had multifocal DCIS. Most women (96%) had no co morbidities (Table 1).

**Table 1:** Patient characteristics

	**N = 1524**
**Age**	
Mean (SD)	57.1 (12.1)
<45	248 (16.3%)
45–50	243 (15.9%)
>50	1033 (67.8%)
**Nuclear grade**	
Low	99 (6.5%)
Intermediate	286 (18.8%)
High	426 (2.08%)
Unreported	713 (46.8%)
**Resection Margin Width**	
≤2 mm	668 (43.8%)
>2 mm	436 (28.6%)
Unknown	420 (27.6%)
**Multifocality**	
Present	340 (22.3%)
Absent/Unreported	1184 (77.7%)
**Necrosis**	
Present	618 (40.6%)
Absent/unreported	906 (59.4%)
**Histologic Subtype**	
Solid	833 (54.7%)
Cribriform	256 (16.8%)
Other	107 (7.0%)
Unreported	328 (21.5%)
**Axillary Node Dissection**	
Yes	585 (38.4%)
No	939 (61.6%)

*N* number of patients, *SD* standard deviation

After mean follow-up of 10.2 years, 36 patients (2.4%) developed chest wall recurrence (17 were DCIS, 19 were invasive cancer). The 5- and 10-year actuarial rates of any chest wall recurrence-free survival were 98.4 and 97.5%. The 5- and 10-year actuarial DCIS recurrence-free survival rates were 99.2 and 98.9%, and invasive chest wall recurrence-free survival rates were 99.2 and 98.6% respectively. The 10-year contralateral breast cancer-free survival rate was 94.0%. The 10 year breast-cancer specific survival rate was 95.9% and the 10-year overall survival rate was 86.5%.

Individuals who developed an invasive chest wall recurrence had a significantly higher risk of dying of breast cancer compared to those who did not develop an invasive recurrence. At 10 years, the cumulative rate of breast cancer mortality was 40.3% for patients who developed invasive recurrence, 23.5% for individuals who developed recurrent DCIS and 3.6% for those who had no recurrence (Gray test p < 0.0001).

We examined the features of women who developed chest wall recurrence in an effort to identify factors associated with an increased risk of chest wall recurrence following mastectomy for pure DCIS. Individuals who developed chest wall recurrence were more likely to be younger than 45 years at diagnosis (19.4 vs. 16.2%; p = 0.20), have high nuclear grade DCIS (36.1 vs. 27.8%; p = 0.41), or close resection margins (52.8 vs. 43.6%; p = 0.23) but these differences did not achieve statistical significance (Table 2).

**Table 2:** Patient characteristics and chest wall recurrence following mastectomy for DCIS

	**Chest Wall Recurrence**		
**Variable**	**No (N = 1488)**	**Yes (N = 36)**	**p value**
**Age**			
Mean (SD)	57.2 (12.1)	54.1 (11.3)	0.13
<45	241 (16.2%)	7 (19.4%)	0.20
45–50	236 (15.9%)	7 (19.4%)	
>50	1011 (67.9%)	22 (61.1%)	
**Nuclear Grade**			
Low	98 (6.6%)	<5	0.41
Intermediate	282 (19.0%)	<5	
High	413 (27.8%)	13 (36.1%)	
Unreported	695 (46.7%)	18 (50.0%)	
**Resection Margin Width**			
≤2 mm	649 (43.6%)	19 (52.8%)	0.23
>2 mm	429 (28.8%)	7 (19.4%)	
Unknown	410 (27.6%)	10 (27.8%)	
**Multifocality**			
Absent/Unreported	1154 (77.6%)	30 (83.3%)	0.41
Present	340 (22.4%)	6 (16.7%)	
**Necrosis**			
Present	604 (40.6%)	14 (38.9%)	0.74
Absent/unreported	884 (59.4%)	22 (61.1%)	
**Histologic Subtype**			
Solid	806 (54.2%)	27 (75.0%)	0.07
Cribriform	252 (16.9%)	<5	
Other	107 (7.2 %)	<5	
Unreported	323 (21.7%)	<5	
**Node Dissection**			
Yes	574 (38.6%)	11 (30.6%)	0.33
No	914 (61.4%)	25 (69.4%)	

*N* number of patients, *SD* standard deviation

On univariate (and multivariable) analysis, none of the factors including resection margin width (univariate HR: ≤2 mm = 2.1 (95% CI 0.86–5.1), p = 0.10), age at diagnosis (age < 45 years, HR = 1.29 (95% CI 0.55–3.02); p = 0.72), high nuclear grade (univariate HR = 3.0 (95% CI 0.4–23.1), p = 0.29), subtype, presence of multifocality (univariate HR = 0.7 (95% CI 0.28–1.62), p = 0.36), presence of comedo necrosis (univariate HR = 1.7 (95% CI 0.23–13.1), p = 0.60) or breast surgeon volume (univariate HR for low volume = 2.6 (95% CI 0.89–7.37), p = 0.08) were associated with an increased risk of chest wall recurrence (Table 3).

**Table 3:** Predictors of chest wall recurrence after mastectomy for DCIS: Univariate analysis

**Parameter**		**Reference**	**Chest wall recurrence** **HR (95% CI)**	**p value**
Age	Continuous	-	0.98 (0.95-1.01)	0.19
	<45	>50	1.29 (0.55-3.02)	0.72
	45-50	>50	1.3 (0.58-3.16)	0.7
Nuclear Grade	High	Low	3.0 (0.4-23.1)	0.29
	Intermediate	Low	1.4 (0.16-12.60)	0.76
	Unreported	Low	2.3 (0.31-17.53)	0.41
Margin Width	≤2 mm	>2 mm	2.1 (0.86-5.1)	0.10
	Unreported		1.59 (0.54, 4.37)	0.35
Multifocality	Present	Absent	0.7 (0.28-1.62)	0.36
Necrosis	Present	Absent	1.7 (0.23-13.1)	0.60
Histological Subtype	Cribriform	Solid	0.5 (0.17-1.37)	0.16
	Other	Solid	0	0.98
	Unreported	Solid	0.48 (0.18-1.24)	0.13
Breast Surgeon Volume (quintile)	1	5	2.6 (0.89-7.37)	0.08
	2	5	1.1 (0.31-3.70)	0.91
	3	5	1.80 (0.59-5.49)	0.30
	4	5	1.4 (0.43-4.26)	0.61
Year of Diagnosis	1997-1999	1994-1996	0.60 (0.28-1.29)	0.13
	2000-2002	1994-1996	0.43 (0.19-1.00)	0.13

*CI* confidence interval, *HR* hazard ratio

**Figure 1:**
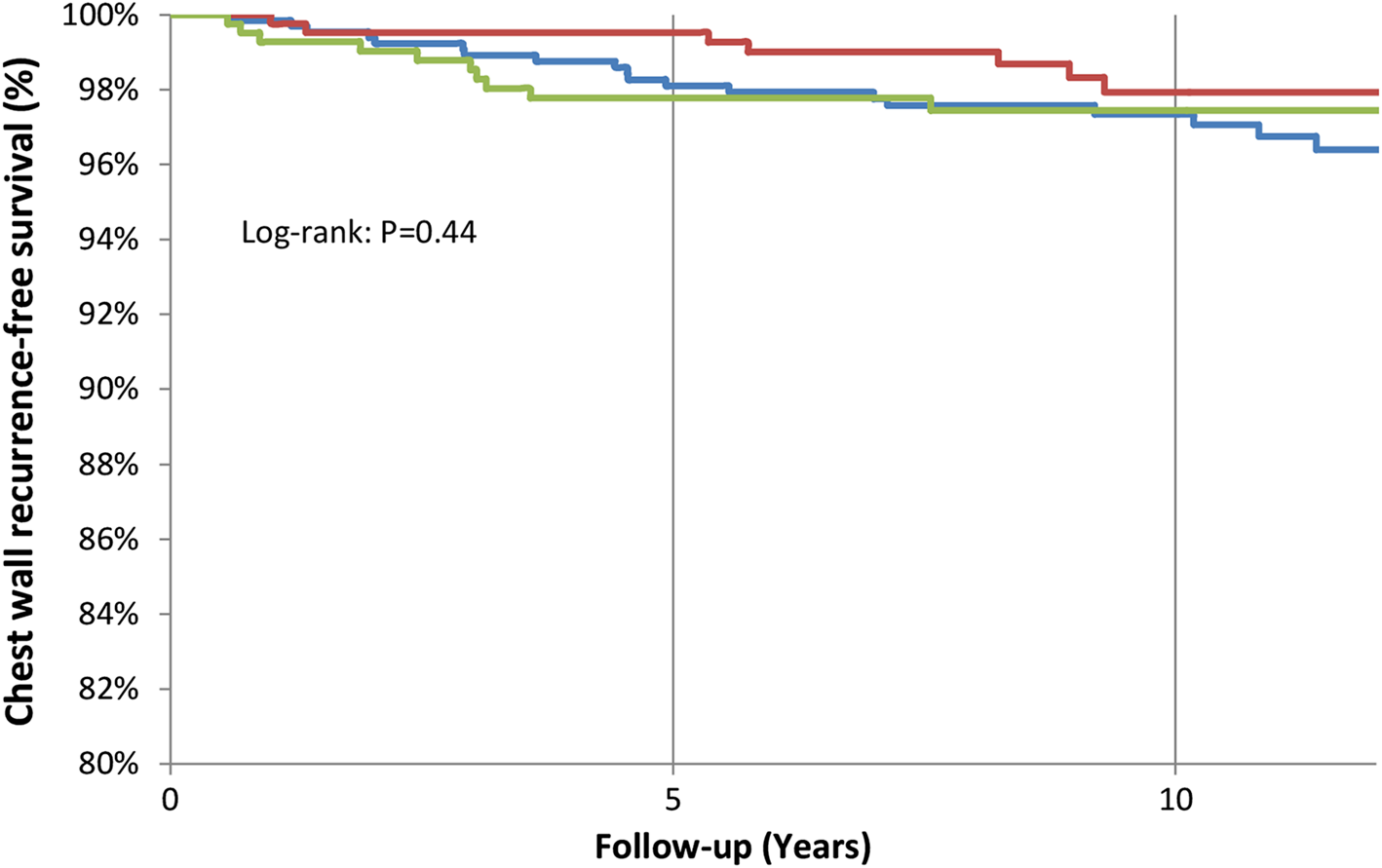
Kaplan–Meier curve showing chest wall recurrence-free survival in women with pure DCIS treated with mastectomy divided into subgroups by resection margin status. *Blue* close (≤2 mm) margins, *Red* negative (>2 mm) margins, *Green* unreported margin status.

We evaluated the 10 year actuarial rates of chest wall recurrence among subsets of individuals with adverse features of DCIS. We did not observe an increased rate of chest wall recurrence among individuals with close (≤2 mm) resection margins, young age at diagnosis or high nuclear grade. The 10-year actuarial rate of chest wall recurrence was 2.7% (19/668) for those with margins ≤2 mm and 2.1% (7/436) for those with wider (>2 mm) negative margins (p = 0.24). The Kaplan–Meier curve illustrating actuarial chest wall recurrence-free survival in subgroups divided by margin status is shown in Figure 1; no difference was seen between the groups with close (≤2 mm), negative (>2 mm) or unreported surgical margins (p = 0.44). Furthermore, the 10 year rate of chest wall recurrence was 1.4% among women with margins ≤1 mm, and 1% for those with positive resection margins (p = 0.33).

Among women younger than 45 years at diagnosis, the 10-year actuarial rate of chest wall recurrence was 2.7% (7/248), 2.9% (7/243) for those aged 45–50 years compared to 2.4% (22/1033) for those older than 50 years (p = 0.2). The 10-year actuarial rate of chest wall recurrence was 3.5% (13/426) among women with high grade DCIS, ≤5% (≤5/286) for those with intermediate grade DCIS, 2.5% (18/713) for those with unreported grade and ≤5% (≤5/99) for those with low grade disease (p = 0.44). We then evaluated the outcomes of those with several adverse features. Among 130 women younger than 45 years at diagnosis with close resection margins (≤ 2 mm), the 10-year actuarial rate of chest wall recurrence was < 5%. Among 83 women younger than 45 years with high grade DCIS, the 10 year actuarial rate of chest wall recurrence was <5%. Among 202 women with high grade DCIS and margins ≤2 mm, the 10 year actuarial rate of chest wall recurrence was 3.2% (Table 4).

**Table 4:** Chest wall recurrence rates by patient subgroup

**Variable**	**N**	**Chest wall recurrences**	**10 year actuarial chest wall recurrence rate**	**p value**
**Age**				0.20
<45	248	7	2.7	
45–50	243	7	2.9	
>50	1033	22	2.4	
**Nuclear Grade**				0.44
Low	99	<5	<5%	
Intermediate	286	<5	<5%	
High	426	13	3.5	
Unreported	713	18	2.6	
**Margin Width**				0.24
≤2 mm	668	19	2.7	
>2 mm	436	7	2.1	
Missing	367	10	2.9	
**Multifocality**				0.37
Present	340	6	2.7	
Absent	1184	30	1.9	
**Necrosis**				0.80
Present	618	14	2.5	
Absent	906	27	1.3	
**Histologic Subtype**				0.08
Solid	833	27	3.6	
Cribriform	256	<5	<5	
Other	107	<5	<5	
Unreported	328	<5	<5	
**Year of Diagnosis**				
1994–1996	378	15	4.0	0.1
1997–1999	499	12	2.4	
2000–2002	647	9	1.4	
**Combined factors**				
Age <45 years + high grade grgrade	83	<5	<5	
Age < 45 years + margins <2 mm	130	<5	<5	
High grade + margins <2 mm	202	6	3.2	

## Discussion

It is well documented that factors such as young age at diagnosis, presence of high grade DCIS and close or positive resection margins are associated with an increased risk of local (in-breast) recurrence following breast-conserving therapy for DCIS, but it remains unclear if women with these features experience an increased risk of chest wall recurrence after mastectomy (Bijker et al. 2001; Dunne et al. 2009; Van Zee et al. 1999; Vicini et al. 2000; Tunon-de-Lara et al. 2001). We evaluated the long-term rates of chest wall recurrence in a large population cohort of women with pure DCIS treated by mastectomy alone (without radiotherapy). The cumulative 10 year rate of chest wall recurrence was 2.4%. We did not find a significant increased risk of chest wall recurrence associated with resection margin status, nuclear grade or age at diagnosis.

Past studies, summarized in Table 5, corroborate our findings of low rates of chest wall recurrence after mastectomy for pure DCIS (Carlson et al. 2007; Owen et al. 2013; Chadha et al. 2012; Fitzsullivan et al. 2013; Meijnen et al. 2008; Godat et al. 2009; Rashtian et al. 2008; Chan et al. 2011; Childs et al. 2013; Kelley et al. 2011; Silverstein et al. 1995; Spiegel and Butler 2003; Vargas et al. 2005) but the reported impact of close or positive resection margins on the risk of chest wall recurrence is less consistent. Chadha et al. (2012) reported the outcomes of 214 women with DCIS treated by mastectomy with median follow-up of 4.6 years; 2 of 24 (8%) cases with margins ≤1 mm developed chest wall recurrence compared to none of 187 women with wider resection margins (p = 0.013). Another study including 80 women with DCIS treated by mastectomy with resection margins <10 mm with median follow-up period of 82.3 months, reported that 5 of 31 women (16%) with margins ≤2 mm developed chest wall recurrence compared to 1 of 49 (2%) with wider margins (p = 0.04). However, data derived from these studies are limited by the inclusion of small numbers of patients from a single institution with few chest wall recurrences and short follow-up intervals (Carlson et al. 2007; Chadha et al. 2012; Rashtian et al. 2008).

**Table 5:** Studies reporting the impact of close or positive resection margins on chest wall recurrence after mastectomy for DCIS

	All Patients in Study			Close or Positive Margins			
Study (Author, Year)	Median follow-up	N	CWR rate	Margin definition	N	CWR rate	p value
Current	10.1 y	1546	2.3%	Positive	305	3.0%	0.24
				≤2 mm	220	1.4%	0.84
Childs, 2013 [14]	7.6 y	142	1.4%	positive	21	4.8%	
				≤2 mm	23	4.3%	
Owen, 2013 [6]	12 y	637	1.9%	Positive	31	6.2%	NS
				<2 mm	35	3.6%	NS
Fitzsullivan, 2013 [8]	6.3 y	803	1%	<1 mm	59	5.0%	<0.001
Chadha, 2012 [7]	4.6 y	211	0.9%	<1 mm	24	8.3%	0.013
Chan, 2011 [13]	8 y	193	NR	Positive	4	0	
Godat, 2009 [11]	4.5 y	83	1.1%	<2 mm	20	0	
Rashtian, 2008 [12]	61 m	80	7.5%	<2 mm	31	16.1%	0.036
Carlson, 2007 [5]	82.3 m	223	5.1%	<1 mm	19	10.5%	0.32
Spiegel, 2003 [17]	10.5 y	44	0	<1 mm	6	0	NS

*CWR* chest wall recurrence, *m* months, *N* number of patients, *NR* not reported, *NS* not statistically significant, *y* years

A larger single institutional study of 803 patients with median follow-up of 6.3 years found an increased risk of chest wall recurrence (5.0%) among 59 women with margins ≤1 mm compared to a rate of 3.6% among 35 women with margins 1.1–2.9 mm and 1% among 744 women with wider negative resection margins (≥3 mm) (p = 0.005) (Fitzsullivan et al. 2013). Another population-based study included 637 women with DCIS treated by mastectomy with median follow-up interval of 12 years (Owen et al. 2013). The authors reported a 6.2% rate of chest wall recurrence among 31 women with positive margins compared to 3.6% for those (N = 35) with close (≤2 mm) margins and 1.5% for those with wider negative margins, but the differences were not statistically significant. In our population, the 10 year rates of chest wall recurrence were 2.7% among 668 women with close margins ≤2 mm, 1.4% among women with negative margins ≤1 mm, 1% among women with positive resection margins and 2% for 436 women with margins >2 mm (p = 0.33). Overall, the data do not suggest that the presence of close or positive resection margins after mastectomy is associated with a significant risk of chest wall recurrence such that routine chest wall radiotherapy is warranted.

We did not find an increased risk of chest wall recurrence among individuals with high grade DCIS. Past studies report rates of chest wall recurrence among individuals with high grade DCIS treated by mastectomy range from 1.5 to 6.4% at 5–10 years (Owen et al. 2013; Fitzsullivan et al. 2013; Chan et al. 2011). Rashtian et al. reported a higher rate of chest wall recurrence among 16 patients with high grade DCIS and margins ≤2 mm (25%) compared to 3% for 64 patients without either risk factor (p = 0.0055) after median follow-up of 61 months.(12) In our population cohort, the 10 year rates of chest wall recurrence was 3.0% among 426 patients with high grade disease, and <5% among 286 with intermediate grade DCIS and <5% for 99 cases with low grade DCIS (p = 0.44). Among 202 women with high grade disease and close (≤2 mm) negative margins 6 developed chest wall recurrence corresponding to a 10-year actuarial recurrence risk of 3.2%.

Some studies report an increased risk of chest wall recurrence among young women with DCIS treated by mastectomy (Owen et al. 2013). In one study of 55 women younger than 40 years at diagnosis the 10 year rate of chest wall recurrence was 7.5% compared to 1.5% for older women (p = 0.003). Overall, published studies report 10 year rates of chest wall recurrence ranging from 0 to 7.5% at 5–10 years among younger women with DCIS treated by mastectomy (Carlson et al. 2007; Owen et al. 2013; Chan et al. 2011; Childs et al. 2013). Our population cohort included 248 women age <45 years at diagnosis; the 10-year rate of chest wall recurrence was 2.7% similar to the rate of 3% for those 45–50 years and 2.4% for women older than 50 years (p = 0.20). Among 130 women <45 years at diagnosis with close (≤2 mm) margins, the 10 year rate of chest wall recurrence was <5%. For 83 women <45 years with high grade DCIS, the 10 year rate of chest wall recurrence was <5%. Overall, we did not find a higher risk of recurrence in younger women. Further data on the outcomes of younger women are needed to determine if younger women have an increased risk of chest wall recurrence.

The strength of this study is that it reports the outcomes of a large population-based cohort of women with DCIS treated by mastectomy with long-term follow-up (median 10.2 years). Individuals who developed chest wall recurrence without a surgical excision or biopsy for tissue confirmation of the chest wall recurrence would not have been identified; therefore, the cumulative rate of chest wall recurrence (with or without simultaneous distant metastases) may be underestimated. Chest wall radiotherapy is associated with a risk of acute and late toxicity, including a detrimental effect on cosmesis following breast reconstruction. Therefore, we aimed to identify baseline factors associated with the development of isolated chest wall recurrence in women with pure DCIS treated by mastectomy, in an effort to identify the subset of individuals who might benefit from post-mastectomy adjuvant radiotherapy. We did not identify any feature, alone or in combination, that was associated with a significant increased risk of chest wall recurrence (at 10 years of follow-up) such that routine radiotherapy should be recommended. However, several pathology reports did not contain information on all pathological features. Lack of completeness in pathological reporting of DCIS has been previously described (Rakovitch et al. 2004; Staradub et al. 2002). Improved adherence will facilitate the impact of pathological features on risks of recurrence.

## Conclusions

Individuals with pure DCIS treated by mastectomy experience low rates of chest wall recurrence. We did not identify a subset of patients, including those with close or positive resection margins, who experienced a high rate of chest wall recurrence.
